# Experiences and challenges of refugees from Ukraine in accessing healthcare and social services during their integration in Lithuania

**DOI:** 10.3389/fpubh.2024.1411738

**Published:** 2024-10-21

**Authors:** Rita Urbanavičė, Rabie Adel El Arab, Vaiva Hendrixson, Donatas Austys, Agnė Jakavonytė-Akstinienė, Marija Skvarčevskaja, Natalja Istomina

**Affiliations:** ^1^Faculty of Medicine, Vilnius University, Vilnius, Lithuania; ^2^Faculty of Nursing and Physiotherapy, University of Lleida, Lleida, Spain; ^3^Healthcare Research Group (GRECS), Institute for Biomedical Research (IRBLleida), Lleida, Spain; ^4^Almoosa College of Health Sciences, Al-Mubarraz, Saudi Arabia

**Keywords:** Ukrainian refugees, healthcare access, social services, language barriers, integration, Lithuania, refugee health

## Abstract

**Background:**

The influx of Ukrainian refugees to Lithuania due to the ongoing conflict has created substantial challenges in healthcare and social services.

**Objective:**

To identify the key challenges faced by Ukrainian refugees in accessing healthcare and social services in Lithuania.

**Methods:**

A qualitative study was conducted using semi-structured interviews with 22 Russian-speaking female Ukrainian refugees residing in various regions of Lithuania. Data were collected between July and October 2022 and analyzed using inductive qualitative content analysis to identify key themes related to healthcare access, social services, and integration.

**Findings:**

Refugees reported significant barriers, including language difficulties, long waiting times for healthcare services, and inconsistent access to social services, particularly in smaller municipalities. Despite access to free healthcare, the quality and timeliness of services were often inadequate, exacerbating challenges for those with pre-existing health conditions. Psychological support services were underutilized, and language barriers impeded access to both healthcare and employment opportunities. Coordination between social and healthcare services was lacking, creating further difficulties for refugees in navigating essential services.

**Conclusion:**

While Lithuanian authorities have provided critical support, significant gaps remain in healthcare access and social service coordination. Urgent improvements are needed in language support, psychological care, and healthcare accessibility, particularly in smaller municipalities. These findings highlight the need for targeted policies to address these challenges and ensure equitable access to services for all refugees. Future research should include more diverse refugee populations to guide comprehensive policy development.

## Introduction

Since the outset of the Russian Federation’s military offensive in Ukraine on February 24, 2022, over 6.2 million people have sought refuge outside the country in 2022 (1). This represents the largest displacement of people in Europe since World War II (2). As of November 1, 2023, nearly 82 thousand Ukrainian citizens have arrived in Lithuania, with this number steadily, albeit marginally, increasing. Most Ukrainians registered their residence in Lithuania’s largest cities. This accounts for approximately 69% of the refugee population in Lithuania. Up to this date, approximately 62% of the Ukrainian refugees were of working-age (18–64 years old), children under 18 took about 33%, and those over 65 years old took about 5%. Notably, the war situation prevented males aged 18–60 from leaving Ukraine, resulting in a predominance of female refugees (3). Among the refugee population, there were representatives from 33 occupational groups, encompassing a total of 2,567 different occupations. Remarkably, 56% of working-age individuals between 18 and 64 years old were employed in Lithuania, as reported by the Ministry of Social Security and Labour of the Republic of Lithuania in 2023 (4).

Huge migration flows created a huge demand for health services and the need to monitor and control them (5). Migrants might have different health care needs, preferences and expectations, also they might face with discrimination, financial problems, loss of family and community, limited access to social and educational services and unclear migration procedures (6), as well as cultural (7, 8), and religious differences (7). Refugees often have chronic illnesses, physical and mental health disorders (9), such as depression, anxiety disorder, post-traumatic stress disorder (10–13), bipolar disorder, and schizophrenia, an acute stress disorder, caused by witnessing Russian attacks, insufficient financial resources, and feeling of loneliness in the host country (13). Health services are a significant requirement for refugees (14), but most refugees arrive without their own health records or sufficient medication, and treatment varies from country to country (15). Age, gender, marital status, employment, English proficiency, social status, social support within and outside the family, leisure time, length of stay in the host country, sense of belonging to the community, and satisfaction with the place of residence (16), income (17, 18) were found to be important social determinants of refugees’ mental health.

Migration should be considered as a key social determinant of health (19), taking into account post-migration factors, such as integration, as predictors of health outcomes among refugees (20, 21). Difficulties experienced during integration, such as bureaucratic barriers and social disengagement, might negatively affect health (20, 22). Improving each of the social determinants of health therefore plays an important role in improving the health of these people (16).

The health system challenges faced by states are relevant to all countries hosting war refugees. This vulnerable population should be taken into account for both immediate and long-term healthcare needs (15). The Social Determinants of Health provide insights into how political and socio-economic policies affect the health and social needs of migrants (23). The participants’ experiences and the challenges they faced are intertwined with various factors that influence the Social Determinants of Health. In this study, we asked the research questions: what are the needs and expectations of refugees upon arrival in the host country; how do the cultural and language barriers affect their integration into the society; what are the refugees’ experiences with healthcare and social services; what are the problems and associated key factors as a perceived by refugees; what are the economics factors affecting their satisfaction with their integration.

### Aim

This study aimed to identify the key challenges faced by Ukrainian refugees in accessing healthcare and social services in Lithuania.

## Materials and methods

### Study design

This study is a part of the research project titled “Assessing the health and social service needs of asylum seekers and refugees; Cultural Competence of Nurses” (24, 25). The qualitative research method, semi-structured interview, was chosen. The interview method enables a deeper exploration of the problems, revealing the experiences of the participants. The interview guide was developed based on existing literature and internal discussions in English. It was translated into Russian and back-translated by two bilingual speakers. During the interviews, socio-demographic characteristics of the respondents were registered. Respondents were asked about their expectations upon arriving in Lithuania, their perceptions of both the positive and negative aspects of life in Lithuania, their awareness of healthcare services and their knowledge of Covid-19, their experiences in seeking treatment for health issues in Lithuania, their observations of cultural differences between Lithuania and their home country, and the impact of these disparities. Additionally, participants were asked about their experiences learning the Lithuanian language, their prospects for employment or education, their living conditions, changes in their leisure activities, their social network of close acquaintances, and their opportunities for integration into Lithuanian society.

### Sample

The study employed a purposive sampling method combined with snowball sampling to select participants from the study population. Eligible participants were refugees who had arrived in Lithuania from Ukraine since February 24, 2022. This study included respondents who had come to Lithuania either due to the conflict initiated by the Russian Federation or had arrived prior to February 24, 2022, and were unable to return to their homes because of the ongoing war. Also, all participants of this study were over 19 years old, female, and had resided in various regions of Lithuania for more than four weeks at the time of the interview.

In this study, saturation was achieved after conducting 22 semi-structured interviews with Ukrainian refugees.

The decision to limit the sample to 22 participants was driven by several factors. First, during data analysis, after approximately 18–20 interviews, the research team observed a repetition of key themes and no new insights were emerging from subsequent interviews. This is a strong indication that thematic saturation had been reached. Conducting additional interviews would have likely led to data redundancy rather than new, valuable insights.

Second, the sample size of 22 was deemed adequate for achieving a deep understanding of the specific challenges faced by the target population. In qualitative research, particularly in studies with narrowly defined populations, smaller sample sizes can be sufficient to uncover meaningful themes, especially when the goal is to explore experiences in depth rather than generalize findings to a broader population. The combination of purposive sampling and the clear emergence of recurring themes confirmed that the sample size was appropriate for this study’s scope and objectives.

### Data collection

The interviews with Ukrainian refugees were conducted from July 30, 2022, to October 19, 2022, at various locations. These interviews were held either at the university, in Ukrainian accommodations, or at another public venue such as a café or park, as preferred by the participants. All the interviews were conducted in the Russian language. There were no participants who would express any discomfort or hindrance in communication in Russian throughout the interview process. During the interviews, with the participants’ prior approval, we utilized the mobile phone application Voice Memos to record the conversations. In cases where participants declined the recording of their interviews, we obtained their consent to transcribe the interview content into text form for analysis. Participants’ agreement on the content of the written text was obtained. On average, each interview had a duration of approximately 30 min.

### Data analysis

The audio recordings obtained during the interviews were transcribed using Microsoft Word software. All the data obtained during this study were coded and the names of the persons named during the interviews were encoded to ensure confidentiality and anonymity. The data obtained from the interviews with the Ukrainians were analyzed using inductive qualitative content analysis with open coding, clustering, categorization, and abstraction (26), where no explicit theory is imposed on the data, and specific hypothesis testing.

### Ethical consideration

The design and completion of this study were guided by the Declaration of Helsinki (27), the Guidelines for the Assessment of Compliance with Research Ethics approved by the Office of the Ombudsman for Academic Ethics and Procedures of the Republic of Lithuania, as well as the following ethical principles: trustworthiness, integrity, respect, and accountability (28). This study has been approved by the Ethical Committee of the Department of Nursing of the Institute of Health Sciences of the Faculty of Medicine of Vilnius University (24.3.2022, no. (1.3)150000-KP-47, supplemented on 11.5.2022, no. (1.3)150000-KP-69, 30.1.2023, No. KT-39). Prior the interview every participant received a comprehensive information sheet and consent form. All the participants were informed that their involvement in the research was entirely voluntary and that they had the option to withdraw their participation at any time. Interviews were conducted (including recording of the interviews) only with the explicit consent of the respondents.

## Results

The participants were Russian-speaking women residing in the city, totaling 22 in number. Among them, there were more participants aged 40 and above (*n* = 12) than those aged 39 and under (*n* = 10), with a mean age of 41 (ranging from 19 to 64). A majority of the participants (*n* = 13) held a university degree, were either married or in a civil partnership (*n* = 14). Nearly all participants (*n* = 21) had children. Only a small proportion of participants (*n* = 8) were proficient in English. Half of the participants (*n* = 11) reported having a health impairment. The majority of the respondents (*n* = 19) were employed in their home country. Half of the participants (*n* = 12) indicated income lower than 500 USD, much smaller part of the sample (*n* = 6) reported incomes ranging from up to 1,000 USD to over 1,500 USD (*n* = 4) in their home country. Most of the participants (*n* = 14) resided in self-rented accommodation in Lithuania (Table 1).

**Table 1:** Characteristics of participants (*n* = 22).

Variable		Number of participants	Identification code of participant
Gender	Female	22	P1-P22
Age	39 years and younger	10	P4, P6, P8- P9, P11, P13-P16, P18
	40 years and older	12	P1-P3, P5, P7, P10, P12, P17, P19-P22
Residential place in their home country	Urban area	22	P1-P22
Educational level	Non-university (High school, College)	9	P5, P8-P9, P11-P12, P18, P20-P22
	University	13	P1-P4, P6-P7, P10, P13-P17, P19
Marital status	Married/lives with partner	14	P2-P9, P12-P16, P18
	Single	8	P1, P10-P11, P17, P19-P22
Children	Yes	21	P1-P7, P9-P22
	No	1	P8
Language	English	8	P1-P4, P10, P14-P16
	Russian	22	P1-P22
Health problems	No	11	P1-P4, P8-P10, P12, P14, P20-P21
	Yes	11	P5-P7, P11, P13, P15-P19, P22
	Chronic diseases (diabetes, cardiovascular diseases, etc.)	2	P19
	Oncological diseases	2	P7, P22
	Mental health disorders (anxiety, depression, etc.)	2	P5, P18
	Mobility disorder	1	P6, P15
	Visual issues	4	P6-P7, P13, P17
	Other	2	P11, P16
Employment status	Employed	19	P1-P17, P20-P21
	Unemployed	3	P18-P19, P22
Average monthly after-tax income in their home country	Less than 500 US dollars	12	P1, P4, P8, P11-P13, P17-P22
	501–1,000 US dollars	6	P5-P7, P9, P14-P15
	More than 1,501 US dollars	4	P2-P3, P10, P16
Place of residence in Lithuania	Lithuanian family	4	P5, P20-P22
	Rented accommodation	14	P1-P4, P6-P10, P14-P16, P18-P19
	Social housing	4	P11-P13, P17

### Сhoice of destination country

Lithuania emerged as a preferred choice due to several key factors, including the presence of relatives and family members residing there, its favorable geographical location, free health care services, and the ability to communicate in Russian. Most participants (*n* = 9) hope to return to their country of origin in the near future despite the loss of their home. Only a small number of participants (*n* = 7), mostly with small children, plan to stay in Lithuania.

### Needs and expectations

The essential needs of refugees revolve around ensuring their safety and the safety of their children, gaining access to high-quality healthcare services, fulfilling basic social necessities, and securing education for their children. Interestingly, a significant portion of the respondents held expectations of receiving quality, cost-free healthcare for themselves or their children, particularly those who were already dealing with medical issues before fleeing the conflict; held expectations to ensure the education of their children or to meet essential social needs. As respondent P18 and P4 said, *“I searched the internet for information on countries with free medical treatment for cancer patients* “(P18); *“I expected a timely help upon my arrival – looked for a package of social and medical services”* (P4).

The essential safety, health and social needs of the all participants and their children were met, although it was “*psychologically difficult*” (P20). Participants and their children were provided with free, affordable healthcare [“*I like the fact that people who work and pay taxes have health insurance not only for themselves but also for their children*” (P15)], children *“attend kindergarten*” (P1) or “*will go to the Ukrainian gymnasium in Vilnius from September*” (P1), “*strangers offered free housing, helped them with the paperwork to get social benefits*” (P7). As participant P19 said, “*all expectations were exceeded, because Lithuania provided a lot of everything*.”

### Culture

All respondents reported that they had not observed any cultural differences that might impede successful integration into Lithuania. They expressed a positive view of the Lithuanian mentality, character, cultural traditions, and gender equality: *“Lithuanians do not stigmatize adults or children with severe conditions,* e.g.*, Down’s syndrome; they are regarded like all other children. In Ukraine, such children are disregarded as they are believed to be of no use”* (P15).

### Faith

The difference appeared to be faith. But as respondent P20 stated, “[I have] *no problems with the faith.”* Different confessions of faith, according to respondent P17, make it difficult for children, as *“The teenagers reacted to the religion classes peculiarly: some laughed, others refused to attend the religion classes, some others were scorned and felt bad.”* According to him, in Ukraine church has nothing to do with the school.

### Language barrier

The main barrier to integration in Lithuania was the language barrier: *“Language is necessary to adapt and work in Lithuania”* (P1). Language barriers were also evident when trying to access quality healthcare, when dealing with paperwork for various social services or education, as not everybody in Lithuania speaks Russian, especially young people, and when attending Lithuanian events such as theatre. When asked whether they would like to see more writing in Ukrainian in Lithuania, respondent P2 expressed the belief that “*this would not be preferable, as it might diminish the motivation to learn Lithuanian*.”

It is worth noting that most respondents who identified the language barrier as the primary obstacle to successful integration, and who expressed a desire to integrate into Lithuanian society, have already achieved significant integration milestones. They hold jobs, have a place of residence, and have their children enrolled in Lithuanian kindergartens or schools. The language barrier stood out as the primary impediment to successful integration, especially for respondents seeking employment in fields where proficiency in the Lithuanian language is mandatory, such as lawyers, healthcare workers, and teachers.

### Learning Lithuanian

Most Ukrainians attended Lithuanian language courses. Respondents were divided into two groups. The first group consists of those who have studied and are studying Lithuanian (*n* = 15) because they need Lithuanian for work or plan to stay in Lithuania: “<…> *I plan to stay in Lithuania. It will take years for the country to get back on its feet, and the children need to be given a good foundation and education now*” (P15). The second group consists of people who do not plan to learn Lithuanian because English or Russian is sufficient for communication, or they do not plan to stay in Lithuania.

Summarizing the answers of the respondents, it can be stated that free Lithuanian language courses, except for the courses organized by the Ukrainian Centre, were evaluated negatively, as they were introductory. During the interviews, the need for intensive Lithuanian language courses was highlighted: “*If I were to study, I would like to study intensively, not just to have a certificate of completion, but not to actually pay anything. If I were to spend time on a course, I would want to use my time efficiently*” (P19).

Difficulties in language learning also emerged, such as the difficulty of adapting to the time of the course and translating the language into a third language. Mostly all respondents were working people with young children, and it is difficult to adjust to the time of the course as it is usually organized during the day. According to respondent P17, “*In Germany, Ukrainians study German for five hours a day. Learning the language then opens a whole new range of possibilities for integration. But here in Lithuania you cannot afford it because you have to work and earn money to make a living*.” Successful language learning is hampered by translation into a third language, as not everyone understands Russian well and “*you have to translate into a third language-Ukrainian*” (P9), and by the different spelling/terms of Russian: “*Although the children are Russian-speaking, but the spelling/terms of Russian itself is different, because the children are multi-lingual. This makes it more difficult for children to understand Russian translations because the pronouns do not match*” (P17).

### Socialization

All respondents in Lithuania spent their leisure time with family and friends, enjoying activities such as walks and travel. As expressed by respondents P2 and P14, their leisure activities had largely remained consistent, with only the location changing. The financial limitations had impacted their ability to engage in specific leisure activities that they previously enjoyed: “*I used to go to a tennis club in Ukraine, which I enjoyed, but I no longer have the opportunity here*” (P13).

Respondents typically turn to their relatives living in Lithuania for assistance or support, as reported by eight participants. For those without relatives in Lithuania, they often seek help from the dwelling owners, a group comprising seven individuals. These individuals play a crucial role in helping Ukrainians settle in Lithuania, and in many instances, as described by P6, this represents their primary contact and support network. However, it is essential to acknowledge that not all Ukrainians have a reliable support system or feel comfortable reaching out to those closest to them. Some respondents expressed sentiments such as, “*There is no one in Lithuania I can turn to” (P6), “I hesitate to approach landlords because it makes me uncomfortable*” (P5). Respondents also believe that family separation presents a significant barrier to successful integration, particularly because a substantial number of arrivals are single women with children and older individuals.

The theme of loneliness among Ukrainians emerged caused by the lack of friends, separation from family, limited cultural activities and events in smaller municipalities centers.

### Availability and quality of healthcare services

Participants were positive about the availability and quality of healthcare services. Participants highlighted the availability of free, affordable, and high-quality healthcare services for themselves and their children as a significant advantage. One respondent shared their experience, saying, *“<…> I was prescribed treatment for type II diabetes, received a free glucose meter and pills, and had my foot examined. In Ukraine, there were no tests for diabetes, no meter; they only prescribed pills”(P22).*

Furthermore, a significant portion of the participants praised the clarity of healthcare procedures, noting that “*Everything adhered to established rules, avoiding the chaos sometimes found in other countries*” (P16). They also appreciated the efficient Advance Patient Registration (APR) system, rapid laboratory testing, easy access to CT scans, a diverse selection of medicines, and the safety of pharmaceuticals and medical supplies. Notably, the availability of alternatives to medications, which differ between Lithuania and Ukraine, was seen as a significant benefit. Additionally, participants acknowledged the swift response of emergency care services in primary care and the availability of ambulance teams for home visits.

Participants expressed dissatisfaction with the waiting times for both general practitioner (GP) and specialist appointments. Long waiting periods were frequently reported, with most respondents citing waiting times of up to four weeks for GPs and ranging from one month to six months for specialist appointments. Particularly, participant P1 noted a lengthy waiting time of six months for a neurologist consultation. The problems of health care services in the primary health care were the most frequently: “*Primary health care centers still do not know how to treat us Ukrainians because different outpatient clinics provide different information – I still cannot sign up with a family doctor” (P5).*

Respondents who live in the smaller municipalities raised concerns about several healthcare-related issues, including the potential decrease in access to healthcare services due to the health reform. They expressed worries that if a local healthcare facility were to close or reduce its scope of services, they might not have the option to travel to another city for care. Participant P9 also voiced concerns about the professional competence of some specialists and the quality of medical equipment. Participant noted limitations such as the restricted operating hours of pharmacies, particularly their closure on Sundays, and emphasized the necessity for an on-call pharmacy service within the smaller municipalities. Furthermore, respondent P9 had reservations about the communication dynamics among healthcare professionals. There was a prevailing lack of trust in smaller municipalities doctors, exemplified by a situation where a child was prescribed antibiotics for a cold without any diagnostic tests. This led to additional telephone consultations with their family doctors back in Ukraine, underscoring the trust issues that arose from such experiences.

### Educational organization

Participants prioritize ensuring education for their children as a paramount concern. They understand that even after the conclusion of the war, it will take years for Ukraine to fully recover, underscoring the immediate importance of providing education for their children. However, participants have encountered a significant challenge: the scarcity of Ukrainian classrooms in their area. Participant P14 succinctly stated, “*There are no Ukrainian classrooms available to teach our children in Ukrainian.*” Participants emphasized the necessity for Ukrainian-language education, particularly for children in older grades who face the imminent challenge of transitioning to Lithuanian schools without a strong command of the language. The fear of potential exam failures looms large in this context. The study also revealed that some participants had their children enrolled in both Ukrainian and Lithuanian educational institutions through remote learning. Due to the differing educational systems in the two countries, this arrangement often results in children attending different classes simultaneously, highlighting the complexities faced by these families in ensuring their children’s education.

### Organizing social services

Participants identified the following challenges: short consultations at the Migration Centre with long waiting times, limitation of social assistance due to the lengthy production of the identity, the complicated procedure for disability, the complicated paperwork, the lack of a coordinator in the municipality, the unclear distribution of the food parcels in the municipality. *“The Municipality Social Department [in smaller municipalities] is a disaster. They did not want to process anything or do paperwork. Food parcels were not given out immediately, and we were not informed of being due food parcels until they came for the parcels themselves,”* said respondent P18. Participants also identified the problem of social housing for vulnerable groups as the rented accommodations were expensive and the need for qualified labour.

### Employment

Nearly all survey respondents were currently employed, and those who were not actively working cite reasons such as personal disability, caring for a young child with a disability, or having reached retirement age. Respondents who had middle-income positions in Ukraine expressed satisfaction with their current jobs in Lithuania, including their wages, team dynamics, intelligent systems, automation, and overall working conditions. However, high-end professionals in Ukraine, earning above average or substantial salaries, indicated that they were not satisfied with their current employment situation, particularly in the long term. Challenges in finding more suitable employment opportunities often revolved around language barriers or structural obstacles. One common issue was the difficulty in having their Ukrainian education and specialization recognized in Lithuania. As one respondent noted, there were challenges like “*mismatched surnames*” and “*delays in diploma approvals*” (P17).

### Housing

Respondents who rent separate accommodations express high levels of satisfaction. In contrast, those with lower incomes who rent rooms in dormitories due to financial constraints express dissatisfaction. They find the dormitory rooms to be old, small, dark, and of poor quality. They also perceived them as expensive given the quality and felt uncomfortable sharing facilities with others. Participants highlighted the increased risk of infectious diseases, particularly among children, when living in social housing. However, respondents who had lost their homes are content with any provided housing, as they do not incur fees or utility costs, as expressed by P17. Those living with Lithuanians express comfort and satisfaction, enjoying the benefits of having their own room and ample food, with all necessary conditions provided. On the other hand, some respondents were eager to secure employment and rent their own place, “*feeling uneasy about living rent-free*.” Despite offers to pay rent, they were motivated by the desire for increased space and privacy.

Difficulties encountered by respondents in finding a place to live include financial possibilities [“*I do not have the financial possibilities*” (P11), “*I cannot afford it on my own*” (P17)], high rental prices [“*The price of flats has gone up sharply*” (P10), *<…> because people see how much income I live on*” (P17)], high utility bills, lack of supply [“*I looked for three months*” (P16)], not accepting pets [“*It was difficult because I have a daughter and two dogs*” (P16)]. As respondent P17 said, “*I understand that we did not come to the richest country. I understand that Lithuania has already received a lot of Ukrainians. Other countries that are rich, they also limit their support for Ukrainians. So, I am very happy with everything here*.”

### Information

It was pointed out that, although there was a lot of information available in the media, it was quite difficult to find the right information for a specific case. As respondent P4 said, “*There is much information online, but it is difficult to understand and select the one suiting your case. This leads to problems.”* The need for information on health insurance, social benefits, or employment for minor children was also expressed.

### Financial issues

High living costs – rental prices, “*consuming 80% of the salary* “(P4), utility bills, food and service prices – and low wages hindered respondents’ integration and affected their motivation to stay in Lithuania. “*I do not have the financial possibilities* “(P11); “*I cannot afford it on my own* “(P17), said respondents P11 and P17. Limited finances also affected the quality of participants’ leisure time, participants’ satisfaction with their life in Lithuania.

Participants offered advice on improving health and social services in Lithuania. Most of the suggestions relate to the dissemination of information.

### Enhancing communication

The proposed measures to improve communication included the production of additional leaflets containing contact details (addresses and phone numbers) of various key organizations, the incorporation of a Frequently Asked Questions (FAQ) section in both printed leaflets and on the website, and an expanded dissemination of information about health insurance. Furthermore, enhancing the performance of Registration Centre staff was recommended through increased provision of information to refugees.

### Establishing a hotline

A proposed initiative involved setting up a toll-free hotline, featuring a single telephone number that connects callers with a live operator proficient in Russian, capable of offering assistance regarding social and healthcare matters. This hotline was designed to address a notable gap in services, as highlighted by P19:

“*There was a lack of a live person—a coordinator to provide information. For instance, while leaflets provide phone numbers, there are limited options for Russian speakers. So, where should one turn to? The only shortcoming is that we require an information center where individuals can visit to address their concerns directly, rather than having to navigate complex issues solely from written materials. Some issues necessitate personalized guidance, and no amount of documentation can adequately assist. A single, toll-free telephone number for Ukrainians encompassing all concerns, like a hotline, would enable individuals to call and resolve their essential matters, whether they pertain to medical or social issues. It is vital that this hotline be toll-free, as there may not always be available funds to cover phone charges. Equally important is that the counselor converses in Russian. While not necessarily a specialist, the counselor should possess the capability to guide callers over the phone, directing them to appropriate resources and advising them on the necessary steps to take.*”

### Appointment of municipal coordinators

The appointment of municipal coordinators is crucial, but it is equally important that these coordinators are active and engaged. While some municipalities have coordinators in place, they are often perceived as merely formal.

### Provision of psychological support

Although free psychological services are available, participants expressed a significant demand for increased access to psychological assistance.

### Improving public transport services

Participants who frequently traveled to different cities within the country suggested enhancing the public transport ticketing system. They proposed introducing the option to purchase a single monthly ticket that would be valid for all of Lithuania. Currently, various cities in the country have distinct ticketing procedures, and it is not always feasible to buy a passenger ticket directly from the driver. As articulated by P14:

“*The monthly public transport ticket is not valid in other cities. It could be a separate monthly ticket that could be purchased and used across the entirety of Lithuania. This would eliminate the need to buy separate tickets in each city. Additionally, it would simplify the payment process, which can be complex and unclear when traveling to a different city.*”

### Establishing an educational institutions system

Participants recommended the establishment of a unified website for school enrollment across all schools in Lithuania. As expressed by P14:

*“There is a deficiency of information concerning standardized school admission criteria. A unified website could be established to direct Ukrainian children to schools in different cities that are equipped to accommodate Ukrainian students. Currently, individuals must manually search for information regarding school locations, contact information, admission criteria, and often, the information is available only in Lithuanian or English. In some instances, the websites may not offer comprehensive information, requiring additional inquiries* via *telephone.”*

### Improving the residence declaration system

Participants recommend enhancing Lithuania’s residence declaration system by establishing a unified Lithuanian residence declaration system.

## Discussion

Our research showed the needs and experiences of refugees from Ukraine in their integration in the host country and identified the challenges they faced in accessing health and social services determined by political, structural, and financial factors. The results of the study support the assertion of the Social Determinants of Health model that political systems and social and economic factors determine health (23). Individuals fleeing the war in Ukraine are a subject to a simplified procedure in Lithuania and are eligible for temporary protection for one year, with the possibility of extension through the issuance of a registration certificate or a residence permit by the Migration Department (29). Refugee accommodation includes collective premises provided by the municipality, premises provided by the local population, or a residence chosen by the foreigner. Health insurance ensures free healthcare for all refugees and their children (30). A free social services package was also provided. However, despite the goal of the rapid integration of refugees into Lithuania, refugees faced many challenges in accessing health and social services, and the advice offered by refugees has only highlighted the wight of the challenges.

Our study found that cultural and religious differences did not pose a challenge for refugees. Other studies have found that religious and cultural differences affect migrants’ health (7, 8). Such differences may have been due to the fact that the refugees came from countries that are not very religiously distant from Baltic countries, and to the refugees’ ability to communicate in Russian.

The biggest cultural challenge was language. The language barrier stood out as the primary impediment to successful integration, especially for respondents seeking employment in fields where proficiency in the Lithuanian language is mandatory, such as lawyers, healthcare workers, and teachers. Refugees from Ukraine, aiming for a faster and more financially successful integration in Lithuania, sought intensive Lithuanian language courses, making comparisons with the German language learning strategy, to be able to work according to their profession, and evaluated the Lithuanian language courses organized in Lithuania negatively. This was confirmed by the State Audit Office in 2023. A preliminary investigation report states that 55 percent of Lithuanian language courses for all asylum seekers did not help their integration into the labor market and society, and the municipalities did not even use all the money allocated to them for organizing language courses for refugees from Ukraine (31). Language barrier and language learning are also among the social determinants of health according to the SSSD model. The language barrier and language learning influence mental disorders (16).

Our study suggests that health services were a significant requirement regardless of existing health disorders. Similar findings were observed in other countries (14). The only exception to this trend was Lithuania, where participants’ vulnerability was not differentiated. Refugees arrived in Lithuania with their own health issues or because their children had health problems, prompting them to seek health insurance for free access to healthcare. Among the 17.7 thousand respondents in all refugee households in Ukraine, as many as 22% have at least one person with specific needs. Of these, 13% are older adult individuals who are more susceptible to serious illnesses (37%) or disabilities (21%) (14). Furthermore, it is essential to consider refugees with chronic infectious diseases, as highlighted by the European Centre for Disease Prevention and Control (ECDC) in 2022 and the World Health Organization’s recognition of World Tuberculosis Day. In Lithuania, there has also been observed an overuse of health services, even in the absence of serious health issues, due to the low cost of public services. But other study indicates that migrants often delayed seeking healthcare except in emergency situations (2). Although participants have reported a relatively clear organization of healthcare services, many Ukrainians face challenges in understanding where to obtain health insurance or undergo disability assessments. Participants’ diverse experiences were contingent on the type and quality of services provided. This variance was predominantly influenced by the decisions of local authorities, healthcare location, type, providers, and individual health workers, rather than a coordinated approach. This trend was similar to Slovakia (2).

In Lithuania, the primary barriers to accessing healthcare services were the extended waiting times for appointments with both family doctors and specialists. This dissatisfaction can be attributed to variations in healthcare systems and treatment approaches in different countries, as well as challenges related to the development of primary healthcare centers and the availability of human resources. The long waiting times were substantiated by prior studies (2, 15). The legal framework in Lithuania outlines the patient’s rights, including the ability to choose another healthcare facility, an alternative family doctor, or a different specialist (30). Additionally, there are specific maximum waiting time requirements for receiving healthcare services, such as primary outpatient care, which must be provided within 7 calendar days (32). Considering these issues, it is crucial to provide education on Lithuania’s healthcare system model, waiting list procedures, and patient rights to address these access challenges effectively. This need were confirmed by the suggestions of the respondents.

The findings from our study indicate that there was a wide availability of medicines in Lithuania, but issues arise in smaller municipalities centers due to the absence of pharmacies on duty. Lithuania boasts one of the densest pharmacy networks in the EU, with 47 pharmacies per 100,000 inhabitants, compared to the EU average of 31 pharmacies (33). It is important to note that these issues primarily affect families with young children. The main reason for this problem was that not all Ukrainians have access to means of transportation to reach another city with pharmacies on duty. But in Moldova, a survey of older adult Ukrainian refugees revealed that 28% required emergency medications, including those for conditions such as diabetes, hypertension, and pain relief. However, more than a third of Ukrainians did not have access to the necessary medicines (15). This challenge could be addressed by expanding health literacy and promoting the advance purchase of essential medicines, making them a compulsory addition to every home medicine cabinet.

The theme of loneliness emerged, as refugees feel alone and separated from their spouses and other family members, and the cultural activities and events in smaller municipalities enters were limited. Reducing social exclusion is therefore essential to avoid health inequalities. As well our study identified a pressing need for psychological support among the interviewees, although the participants did not directly acknowledge this need. Instead, they subtly suggested the importance of providing psychological counseling to all individuals. The research suggests that refugees often have depression, anxiety disorder, post-traumatic stress disorder (10–13), bipolar disorder, and schizophrenia, and feeling of loneliness in the host country caused an acute stress disorder (13). For this reason, it is very important to provide psychological support as soon as possible. This is confirmed by participants’ advice to provide psychological support to all refugees.

The basic social needs of the participants were met, but they encountered challenges related to the refugee policies and budgets of individual municipalities. Not all municipalities offered free social housing, distributed food parcels, or provided other complimentary services, such as transportation. Additionally, the level of benefits provided to refugees varied among municipalities. The study also shed light on bureaucratic issues in Lithuania concerning the processing of personal documents, residence registration, and the prolonged document preparation process. The extended production times for personal documents led to the loss of social benefits for participants. These challenges experienced by refugees substantiate that there were significant social inequalities that adversely affected their health, and actions to reduce social exclusion are needed to prevent health inequalities. Similar challenges were observed in other countries hosting refugees (14, 20, 22). These lengthy waiting periods for identity document issuance were not solely attributed to Lithuania’s encounter with a massive influx of migrants for the first time but also stemmed from citizens’ mass applications for identity documents.

The participants often resorted to searching for information they needed on social networks, through online searches, or by seeking advice from acquaintances. Similar situation was in Slovakia (2). However, the study findings indicated that the abundance of information available online posed challenges for participants, as it was challenging to locate the specific information they required. Furthermore, not all relevant information was presented in a language comprehensible to the participants, contributing to their difficulties in finding the necessary information. This could explain why participants encountered difficulties in their search for information. Additionally, participants’ ability to access the required information was influenced by their level of literacy. In contrast to some other countries, the study’s results in Lithuania highlighted that most participants expected to receive essential information with clear explanations at the Migration Centre. However, due to the overwhelming influx of migrants and a shortage of human resources, this need went unmet. Participants’ experiences underscore the significance of having a “live” person available to assist them with various issues in a language they can understand. This need was exemplified by the establishment of a hotline in Germany during a refugee influx (34). It is therefore necessary to consider the suggestions made by refugees, which are mostly related to the dissemination of information.

In Lithuania, participants did not explicitly identified cash as a fundamental need. However, a comparative analysis of content and socio-demographic data suggested that participants’ financial situation significantly influenced their satisfaction with various aspects of their lives, including their employment, income, housing, leisure activities, and other opportunities. Other studies indicated, that financial problems were one of the main integration challenges (6) and indicated that participants emphasized the importance of having access to cash as a fundamental need that enables them to meet their other basic requirements (14). As well financial difficulties were associated with poorer mental health (17, 18).

Most participants (9 out of 22) expressed hope to return to their country of origin which contrasts with the situation in Moldova, Hungary, Belarus, Slovakia, and Romania where up to 63% of the participants plan to remain in their host countries (14). The primary reasons cited for this divergence include patriotism, the high cost of living, low wages, the desire to reunite with family as confirm other study (1). But Participants intending to stay in their host countries express a strong eagerness to integrate quickly, thus emphasizing the importance of intensive Lithuanian language courses. Participants’ future plans serve as a rationale for their relatively passive participation in language courses, their contentment with the services they receive, and the overall success of their integration efforts.

In summary, the participants found the conditions favorable for their integration in Lithuania. The insights and advice shared by the participants have the potential to enhance the reception model for migrants, not only in Lithuania but also in other countries.

### Recommendations

Based on the findings of this study, several actionable recommendations are proposed to improve the healthcare and social services provided to Ukrainian refugees in Lithuania. First, there is an urgent need to enhance psychological support systems, particularly for refugees dealing with trauma-related conditions such as post-traumatic stress disorder, anxiety, and depression. While psychological services are available, outreach efforts are insufficient. We recommend implementing targeted programs that inform refugees about these services through accessible channels, such as bilingual materials in Ukrainian and Russian. Furthermore, community-based mental health programs should be expanded to offer culturally sensitive trauma care, leveraging collaborations with NGOs and international organizations to ensure sufficient resources and training for local psychologists.

In addressing healthcare access, the long waiting times for general practitioners and specialists remain a significant barrier. A fast-track service for refugees with chronic conditions or mental health issues should be introduced, ensuring timely care. Additionally, mobile healthcare units should be deployed in rural areas to ease the burden on urban facilities and decrease wait times. Expanding telemedicine services would also provide quicker access to healthcare for those in remote locations.

Navigating Lithuania’s healthcare system has been identified as a challenge due to language barriers and a lack of accessible information. To address this, we recommend the development of a comprehensive healthcare guide in Ukrainian and Russian, distributed through refugee centers, healthcare facilities, and online platforms. Additionally, appointing bilingual healthcare navigators would assist refugees in understanding and accessing the healthcare services available to them.

Language barriers remain a major obstacle to healthcare access and employment. To mitigate this, the availability of certified medical interpreters should be increased in healthcare settings, and an incentive program for refugees to participate in intensive Lithuanian language courses should be established. These courses should offer financial or job placement support upon completion, helping refugees overcome language challenges more effectively. Healthcare providers should also offer bilingual resources, such as translated patient forms and medication instructions.

Coordination between social services and healthcare providers needs improvement to streamline access to necessary services. Establishing a centralized coordination unit would ensure that social workers are embedded in healthcare facilities, providing comprehensive support for refugees as they navigate healthcare, housing, and employment. Regular interagency collaboration should be prioritized to optimize service delivery across sectors.

For refugee families, particularly women and children, maternal and child health services should be prioritized. Mobile health units or specialized clinics can provide prenatal care, vaccinations, and mental health support for mothers. Collaborations with international organizations can help fund and staff these services, particularly in regions with high refugee populations.

Finally, housing instability poses significant challenges for refugee health and well-being. A public-private partnership should be established to provide affordable, safe housing options, coupled with a housing support fund to help refugees transition into long-term accommodations. This initiative would alleviate overcrowding in temporary facilities and improve overall health outcomes.

These targeted actions can help reduce health inequities and ensure that refugees receive the care and support necessary for successful integration into Lithuanian society.

### Strengths and limitations of this study

The study unearthed certain systemic barriers in the organization of services, which, if addressed, could enhance the quality of services, benefitting not only migrants but also the broader population of the country. Participants had the flexibility to be interviewed in English, Russian, and, in some cases, Ukrainian, depending on their location. This approach ensured that the study remained inclusive, allowing all those who wished to participate to do so without any constraints. However, it is crucial to note that these findings should not be generalized to the entire Ukrainian population. Even though the participants hailed from various regions of the host country, their experiences were diverse and influenced not only by structural and economic factors but also by environmental and personal circumstances. A key limitation of this study is its focus on a homogenous sample of Russian-speaking women from urban areas, which restricts the breadth of the findings. This limited demographic scope may affect the generalizability of the results, as it excludes significant portions of the refugee population—particularly men, rural refugees, and non-Russian speakers—whose experiences may differ considerably in terms of healthcare access, social services, and integration challenges. To broaden the understanding of refugee experiences, future studies should aim to include a more diverse sample, incorporating rural populations, men, and non-Russian speakers, which would provide a more representative view of the challenges faced by Ukrainian refugees as they integrate into Lithuania.

## Conclusion

This study reveals key barriers Ukrainian refugees face in accessing healthcare and social services in Lithuania, with language barriers, long waiting times, and inconsistent service provision being the most significant challenges. While essential needs such as safety and healthcare were met, urgent improvements are needed in psychological support, intensive language training, and healthcare accessibility. Addressing these gaps will reduce health disparities and enhance integration efforts.

The study’s findings are limited by the sample’s homogeneity, focusing solely on Russian-speaking women from urban areas. Future research should explore a more diverse refugee population to inform more inclusive policy responses. Strengthening healthcare and social service coordination is crucial for promoting equitable access and successful refugee integration.

## Data Availability

The original contributions presented in the study are included in the article/supplementary material, further inquiries can be directed to the corresponding author.
